# Model Protocells from Single-Chain Lipids

**DOI:** 10.3390/ijms10030835

**Published:** 2009-03-02

**Authors:** Sheref S. Mansy

**Affiliations:** Department of Chemistry & Biochemistry, University of Denver, Denver, CO 80208, USA; E-Mail: smansy@du.edu; Tel. +1-303-871-2533; Fax: +1-303-871-2254

**Keywords:** Origin of life, prebiotic, vesicle, synthetic biology, fatty acid

## Abstract

Significant progress has been made in the construction of laboratory models of protocells. Most frequently the developed vesicle systems utilize single-chain lipids rather than the double-chain lipids typically found in biological membranes. Although single-chain lipids yield less robust vesicles, their dynamic characteristics are highly exploitable for protocellular functions. Herein the advantages of using single-chain lipids in the construction of protocells are discussed.

## 1. Introduction

Membranes are important cellular constituents, allowing for processes such as ATP synthesis and neurochemical signal-transduction. Due to the long evolutionary history of cellular membranes, they are highly complex assemblies consisting primarily of double-chain lipids, sterols, and transmembrane spanning proteins. This level of complexity is necessary to create an impermeable barrier that allows for both the generation of electrochemical gradients and for the specific transfer of molecules across the membrane. In other words, contemporary cells are capable of maintaining a strong barrier between their internal contents and the extracellular space while retaining the ability to specifically absorb or release desired molecules through the use of transmembrane spanning proteins. The result is an asymmetric, nonequilibrium distribution of molecules that can be harnessed for physiological purposes. Clearly, Earth’s first cell-like structures did not already posses such complexity. Instead, more simple membrane systems likely existed that exhibited many of the characteristics that modern biological membranes possess without relying on genetically encoded transport systems.

Although it is established that membranes are important structures for extant cells, it is not understood at which stage of chemical or early biological evolution were membranes incorporated into precellular systems. Since all known cells exploit lipids of high chemical complexity, an extrapolation to prebiotic membrane compositions is difficult. Furthermore, membranes can facilitate as well as inhibit evolutionary processes. For example, it could be argued that the premature encapsulation of a nascent chemical system would inhibit its growth and development by denying the system chemical nutrients. Conversely, membrane enclosed compartments could capture within its interior unique, non-equilibrium environments necessary for evolution that may not be as easily attainable under equilibrium solution conditions.

Notwithstanding the described difficulties, the concept of simple, amphiphilic molecules spontaneously assembling into vesicular membranes is believed to have been an important early step in the formation of a protocell [1,2]. Membranes provide an organization to chemical components without which demarcation between one chemical mixture from another becomes less defined. This ability to differentiate between one entity and another is a necessary prerequisite for Darwinian evolution. Such spatially resolved compartments not only create an environment for inter-protocellular competition but also facilitate the evolution of intracompartmentalized catalytic molecules, such as a RNA polymerase [3]. The concentration of molecular building blocks can also be facilitated by enclosure within a small volume. For example, one molecule inside of a 100 nm vesicle yields a concentration in the micromolar range, a significantly higher concentration than would be achieved in solution. Therefore, the simple process of encapsulation results in a large concentration effect that can be exploited for chemical reactions, such as the formation of biopolymers. It should be noted that non-lipid defined compartments could provide several of the these described features and are believed by some to be of prebiotic importance [4].

**Figure 1.**
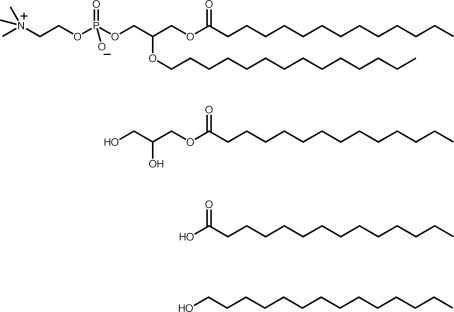
Chemical structures of representative single-chain and double-chain lipids. From top to bottom, the molecules are dimyristoyl phosphatidylcholine (DMPC), monomyristin, myristic acid, and tetradecanol.

What constitutes a prebiotically reasonable lipid? As the events leading to the first cells are not known, it is difficult to evaluate which lipids were prebiotically available. One approach to address this problem is to determine the range of molecules that can be synthesized under simulated prebiotic conditions. For example, such studies have shown that Fischer-Tropsch-type reactions under hydrothermal conditions can produce simple lipid molecules, such as fatty acids [5]. Another approach to provide insight into the types of molecules that can be abiotically synthesized is to analyze the composition of carbonaceous meteorites [6]. This is particularly interesting since organic extracts of meteorite samples were shown by Deamer and colleagues to spontaneously form vesicles in aqueous solution [7,8]. Although this organic extract consisted of a mixture of simple and complex organic molecules, analyses of the Murchison meteorite by Lawless and Yuen revealed the presence of short-chain fatty acids [9]. The identification of fatty acids as products of Fischer-Tropsch-type reactions and their isolation from samples of the Murchison meteorite illustrates the plausibility of the abiotic synthesis of simple lipids. Single-chain lipids, such as fatty acids, are thus attractive constituents of protocellular membranes because they are chemically simple (Figure 1), i.e. prebiotically plausible, and further they readily form bilayer membranes under appropriate solution conditions [10–12], are easily interconvertible between different aggregate structures by changes in pH [13,14], and even show some motility characteristics [15].

## 2. Similarities between fatty acid and phospholipid membranes

Vesicles composed of fatty acids are similar to phospholipid vesicles, which is consistent with the fact that they are both compartments that separate internal and external aqueous environments with a hydrophobic barrier. For example, both fatty acids and phospholipids form vesicles of similar size [12] that are thermally stable [16], are able to retain macromolecules [17], and are capable of withstanding intravesicular protein [17] and RNA [18] mediated enzymatic reactions. Further, both fatty acid and phospholipid vesicles are similar in tensile strength [19], have similar permeability selectivity trends for small, uncharged solutes [20], and generally require acyl-chain lengths of at least 10 carbons to form stable bilayer structures [12,21].

## 3. Differences between fatty acid and phospholipid membranes: lipid hydrophobicity

Despite their similarities, vesicles composed of single-chain lipids are significantly different than double-chain phospholipids. This difference arises, in large part, from the decreased overall hydrophobicity of single-chain amphiphiles, which is manifested as an increase in lipid dynamics and thus a decrease in vesicle stability. The amphiphile dynamics that give rise to such behavior are analogous to those thoroughly described for micelles [22]. More specifically, since single-chain amphiphiles have less overall hydrophobicity than their double-chain counterparts, the activation energy for amphiphile escape from the aggregate structure into solution is lower, and therefore the amphiphile residence time within the aggregate is shorter (since hydrophobicity also correlates with the critical micelle concentration (CMC), residence time can also be related to CMC) [22]. Similarly, the energetic barrier of a single-chain amphiphile, i.e. a less hydrophobic molecule than a double-chain lipid, imbedded in a membrane to rapidly seek thermodynamic equilibrium with its surrounding solution is low, and so single-chain amphiphiles have a short residence time within the bilayer membrane. Therefore, vesicles composed solely of single-chain amphiphiles readily disassemble under low amphiphile conditions. A thorough analysis of fatty acid dynamics of micelles and vesicles has been performed by Walde and colleagues [23–25].

A fascinating example of the utility of such lipid escape dynamics to protocell evolution is its potential to link genotype with phenotype. The encapsulation of ionic polymers yields powerful osmotic pressures beyond that of uncharged macromolecules due to the additional imbalance of associated counterions. The pressure can be partially relieved by vesicle growth. However, facile mechanisms of vesicle growth are not available to membrane systems exhibiting low lipid dynamics, whereas highly dynamic lipid systems are able to acquire additional lipid components for growth. The Szostak group tested a laboratory model of such a system by mixing vesicles with encapsulated RNA molecules with empty vesicles and found that the RNA containing fatty acid vesicles grew by acquiring lipids from the empty vesicles [19]. The implications are that if a replicase, that is a RNA that catalyzes its own replication, were present inside of a fatty acid vesicle, the resulting increased nucleic acid content would cause the vesicle to preferentially grow by acquiring fatty acids from non-replicase containing vesicles [26].

## 4. Differences between fatty acid and phospholipid membranes: lipid flip-flop

**Figure 2.**
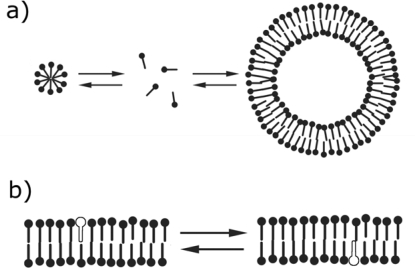
Lipid dynamics. a) Exchange of lipids between different aggregate structures, including micelles (left), free monomers (center), and vesicles (right) b) Lipid flip-flop between inner- and outer-leaflets of a bilayer membrane.

In addition to partitioning effects, the chemical properties of a lipid influences its flip-flop dynamics (Figure 2). Such flipping dynamics are influenced by both the overall hydrophobicity of the lipid and the polarity of the head-group. From this perspective, fatty acids are again more dynamic than their phospholipid counterparts. Phospholipid flip-flop is so slow (t_1/2_ > days) [27] that enzymes exist to flip phospholipids to the desired leaflet, thereby facilitating the attainment of asymmetric lipid distributions within biological membranes. Conversely, the protonated, less polar state of fatty acids flip-flop with a t_1/2_ in the milliseconds [28,29]. Therefore, membranes consisting of fatty acids rapidly equilibrate the compositions of both inner- and outer-leaflets. It is expected that single-chain lipids with more polar head-groups, e.g. phosphate [30,31], would not exhibit such fast flipping dynamics. Vesicles composed of less dynamic lipids could have the advantage of being able to retain pH gradients.

The high degree of flip-flop dynamics allows for several properties advantageous to a protocell. For example, unlike vesicles composed solely of double-chain phospholipids, fatty acid vesicles can grow [32,33] and replicate [32, 34]. This is possible since the dynamics allow for incoming fatty acids to partition into the outer-leaflet of preexisting vesicle membranes and subsequently for the protonated state of the lipid to flip into the inner-leaflet of the membrane [35]. Additionally, fatty acid flip-flop may be responsible for increased membrane permeability. For example, the more dynamic fatty acid membranes are permeable to nucleoside mono- and di-phosphates, whereas the less dynamic phospholipid membranes are typically impermeable to nucleotides [36]. In the absence of transport machinery, such intrinsic permeability would be important for the uptake of critical nutrients. In addition to dynamics, other methods of solute passage have been described. For example, packing defects within membranes of specific phospholipid compositions can be exploited for solute passage [37–39].

## 5. Membranes composed of mixtures of single-chain lipids

It is unlikely that protocellular membranes were composed of a single type of lipid. Instead, they likely consisted of mixtures of lipids. Since lipid compositions influence the physical properties of the resulting membranes, it is important to evaluate the characteristics of how different lipid mixtures modulate membrane properties. Studies into single-chain lipid mixtures revealed a dramatic increase in stability for fatty acid - fatty alcohol mixtures, as evidenced by a decrease in critical vesicle concentration (CVC), an increased ability to withstand alkaline [40], high ionic strength [41], and elevated temperature conditions [16]. The increased stability to basicity is explainable by the lack of hydrogen-bond donors within membranes composed solely of fatty acids above the apparent pK_a_ of the lipid head-group [40]. Similarly, the decrease of metal ligands within the membrane (e.g. by incorporation of fatty alcohols) render the membrane less reactive to metal centers. However, the decreased CVC and the increased stability to temperature is less readily explainable from hydrogen-bond donor - acceptor interactions. Indeed, hydrogen-bonding between two fatty acids is significantly stronger than between a fatty acid and a fatty alcohol. Nevertheless, the CVC and temperature stability data for fatty acid - fatty alcohol mixtures suggest that additional stabilizing influences exist, perhaps due to improved packing between lipids with sterically complementary head group sizes or to decreased charge repulsion between the lipid head-groups. The latter seems to be supported by mixtures of alkyl amines with fatty acids, which have 10-fold lower CVC values [42].

Subsequent studies revealed that the addition of the glycerol monoester of fatty acids to vesicles further increases vesicle stability [18,41,43]. The glycerol monoester head group can participate in two hydrogen-bonding interactions with adjacent fatty acids and thus confers greater membrane stability under alkaline, high ionic strength [18,41], and temperature conditions [16]. A particularly dramatic example is of vesicles composed of 2:1 myristoleic acid and the glycerol monoester of myristoleate (monomyristolein), which are able to withstand Mg^2+^ concentrations of up to 4 mM [18] and temperatures of 100 °C [16]. As many biological processes rely on Mg^2+^ for catalysis [44], the identification of prebiotically plausible lipids capable of surviving moderate concentrations of Mg^2+^ is insightful. Chen *et al*. have used such myristoleic acid:monomyristolein vesicles to encapsulate a functional magnesium dependent ribozyme [18]. The large increase in thermal stability upon the incorporation of monomyristolein provides additional advantages, including increased solute permeability and the ability to thermally separate strands of DNA thus priming the system for genomic replication [16].

A surprising outcome of studies on heterogeneous membrane compositions is their influence on permeability. While admixtures can result in increased stability, they also can simultaneously result in increased permeability. For example, the incorporation of monomyristolein into myristoleic acid vesicles increases ribose permeability 5-fold [36]. The reasons for this appear to be associated with the membrane dynamics of the system. Fatty acids are able to impart permeability to phospholipid membranes by associating with and carrying solutes across the membrane via flip-flop, i.e. by a carrier mechanism [28,45,46]. It therefore follows that membranes composed entirely of lipids that flip-flop quickly should yield more permeable membranes and that the permeability can be further increased by increasing the rate of lipid flip-flop. However, lipid-solute complexes are not the only mechanism by which lipid flip-flop could increase solute permeability. For example, as a lipid flips from one leaflet to the other the lipid passes through an intermediate state in which the hydrophilic head-group passes through the hydrophobic interior of the membrane. The unfavorable proximity of a polar head group with the hydrophobic regions of the surrounding lipids likely results in transient packing defects that could be exploited for solute passage. Perhaps the larger steric bulk of the glycerol head-group of monomyristolein decreases the cohesive interactions between the lipid acyl chains, thus facilitating lipid flip-flop [36].

## 4. Conclusions

Fatty acid vesicles possess dynamic membranes that are highly exploitable for protocellular functions without reliance upon complex protein machinery. Further, mixtures of single-chain lipids can yield enhancements to both vesicle stability and permeability. Thus far, the most characterized lipid mixture is 2:1 myristoleic acid:monomyristolein, which appears to be more conducive to the generation of a protocell than any other tested lipid composition. It will be interesting to observe whether other less investigated lipid mixtures, such as alkyl amines which form membranes that are stable to 0.1 M Mg^2+^ [42], will prove to be more amenable to the construction of a protocell. Further efforts in moving beyond simple stability and permeability mechanisms and into self-sustaining autopoeitic systems, as Luisi and colleagues advocate [47], will undoubtedly move us closer to the laboratory synthesis of tailor-made cells.
